# Supplementary material for: Human EEG and artificial neural networks reveal disentangled representations and processing timelines of object real-world size and depth in natural images
Source: eLife. 2025 Dec 22;13:RP98117. doi: 10.7554/eLife.98117 (PMC12721712; doi:10.7554/eLife.98117)
Supplement: Supplementary file 1. [file elife-98117-supp1.docx]

**Table A** Statistical results of similarities (partial Spearman correlations) between four ANN RDMs and three hypothesis-based RDMs.

| ANN × HYP | ***ResNet (early)*** | ***CLIP (early)*** | ***ResNet (late)*** | ***CLIP (late)*** |
| --- | --- | --- | --- | --- |
| ***Real-World depth*** | **r=.0330, p<.001** | **r=.0262, p<.001** | r=-.0513, p=1 | **r=.0278, p<.001** |
| ***Retinal Size*** | **r=.0618, p<.001** | **r=.0730, p<.001** | **r=.0221, p<.001** | r=.0058, p=.1788 |
| ***Real-World Size*** | r=-.0330, p=1 | r=-.0027, p=.8710 | **r=0.2497, p<.001** | **r=0.2378, p<.001** |

**Table B** Statistical results of similarities (partial Spearman correlations) between four ANN RDMs with inputs of cropped object images without background and three hypothesis-based RDMs.

| ANN (obj-only imgs) × HYP | ***ResNet (early)*** | ***CLIP (early)*** | ***ResNet (late)*** | ***CLIP (late)*** |
| --- | --- | --- | --- | --- |
| ***Real-World depth*** | r=-.0109, p=.9382 | r=-.0086, p=.8862 | **r=.0183, p=.0049** | r=-.0176, p=.9934 |
| ***Retinal Size*** | **r=.0323, p<.001** | **r=.0315, p<.001** | r=-.0032, p=.6725 | r=-.0031, p=.6705 |
| ***Real-World Size*** | r=.0022, p=.3781 | r=.0084, p=.1193 | **r=.0402, p<.001** | **r=.0154, p=.0149** |
